# Tff1-expressing Tregs in lung prevent exacerbation of Bleomycin-induced pulmonary fibrosis

**DOI:** 10.3389/fimmu.2024.1440918

**Published:** 2024-09-02

**Authors:** Masaaki Okamoto, Ayumi Kuratani, Daisuke Okuzaki, Naganori Kamiyama, Takashi Kobayashi, Miwa Sasai, Masahiro Yamamoto

**Affiliations:** ^1^ Department of Immunoparasitology, Research Institute for Microbial Diseases, Osaka University, Suita, Japan; ^2^ Laboratory of Immunoparasitology, World Premier International Research Center Initiative Immunology Frontier Research Center, Osaka University, Suita, Japan; ^3^ Genome Information Research Center, Osaka University, Suita, Japan; ^4^ Department of Infectious Disease Control, Faculty of Medicine, Oita University, Oita, Japan; ^5^ Research Center for GLOBAL and LOCAL Infectious Diseases, Oita University, Oita, Japan; ^6^ Department of Immunoparasitology, Center for Infectious Disease Education and Research, Osaka University, Suita, Japan

**Keywords:** Bleomycin, fibrosis, Treg, Tff1, VeDTR

## Abstract

Bleomycin (BLM) induces lung injury, leading to inflammation and pulmonary fibrosis. Regulatory T cells (Tregs) maintain self-tolerance and control host immune responses. However, little is known about their involvement in the pathology of pulmonary fibrosis. Here we show that a unique Treg subset expressing trefoil factor family 1 (Tff1) emerges in the BLM-injured lung. These Tff1-expressing Tregs (Tff1-Tregs) were induced by IL-33. Moreover, although Tff1 ablation in Tregs did not change the pathological condition, selective ablation of Tff1-Tregs using an intersectional genetic method promoted pro-inflammatory features of macrophages in the injured lung and exacerbated the fibrosis. Taken together, our study revealed the presence of a unique Treg subset expressing Tff1 in BLM-injured lungs and their critical role in the injured lung to ameliorate fibrosis.

## Introduction

Idiopathic pulmonary fibrosis (IPF) is a chronic progressive lung disease of unknown cause with a severe prognosis (1). No curative treatment has been established, and the therapeutic goal is primarily to slow the progression of disease (2). A chronic inflammatory response in the lungs has been observed in IPF, and the immune system is thought to be involved in the pathogenesis of the disease (3, 4). Bleomycin (BLM), a chemotherapeutic agent, is most widely used for rodent models of IPF since its pulmonary toxicity induces inflammation and fibrosis in the lung (5). In the BLM-induced fibrosis model, various types of immune cells including T cells are involved in the disease (6, 7). Among the T cell population, regulatory T cells (Tregs) maintain self-tolerance, suppress host immune responses, and control inflammation (8, 9). Dysfunction of Tregs in IPF has been identified (10), suggesting an antifibrotic role of Tregs. On the other hand, Tregs are known to produce TGF-β, which is a crucial profibrotic cytokine (11, 12), and are also recognized as a profibrotic mediator in IPF (13, 14). Several previous studies demonstrated the role of Tregs in the BLM-induced pulmonary fibrosis model, also raising conflicting claims. Some studies indicate that Tregs ameliorate fibrosis (15, 16) and others indicate that Tregs exacerbate fibrosis (17, 18). These differences might be due to the differences in investigation methods and strategies, but in any case, they suggest the importance of Tregs in the disease state of pulmonary fibrosis.

In this study, to gain a better understanding of the significance of Tregs in pulmonary fibrosis, we perform single-cell RNA-sequencing (scRNA-seq) and Bulk RNA-seq analyses, and reveal the appearance of Tregs uniquely expressing Trefoil factor 1 (Tff1) in the BLM-injured lungs. These Tff1-expressing Tregs (Tff1-Tregs) are induced by IL-33 *in vitro* and *in vivo*. Furthermore, using VeDTR system (19), an intersectional genetic method allowing labeling and removal of Tff1 and Foxp3 co-expressing cells, we demonstrate that conditional depletion of the Tff1-Tregs exacerbates fibrosis. Moreover, Tff1-Tregs play a crucial role in suppressing the inflammatory feature of monocytes and macrophages in fibrotic lungs. Collectively, our study reveals Tff1-Tregs that appear specifically in inflammatory/fibrotic lungs and their significance in suppressing pulmonary fibrosis.

## Results

### Tregs express Tff1 in BLM-injured lung

**Figure 1:**
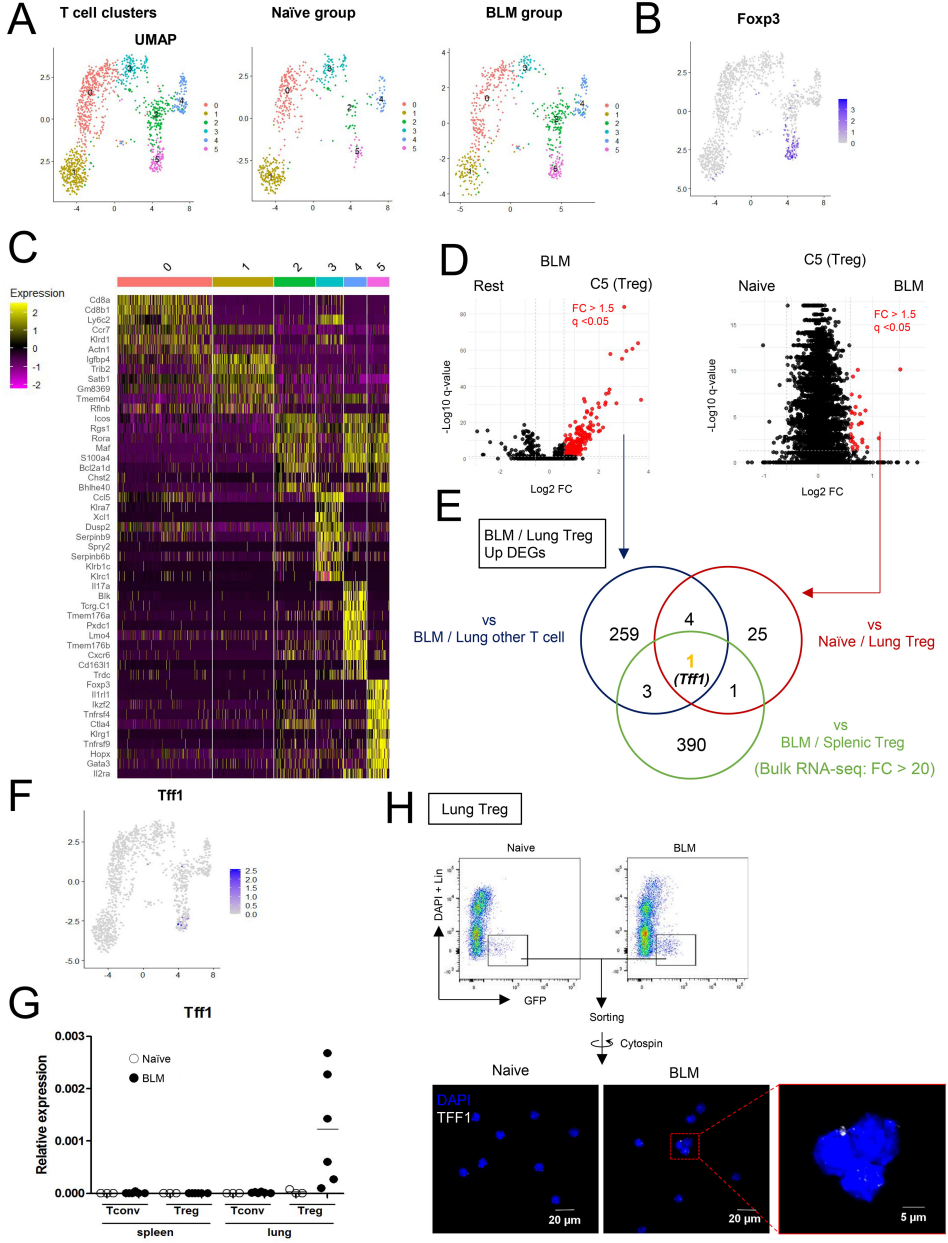
Lung Tregs express Tff1 in BLM-administered lung. **(A–F)** CD45^+^ cells from lungs of naïve and Bleomycin-administered WT mice were applied to scRNA seq analysis. Data shows pooled samples (n = 3 biologically independent mice). See also **Supplementary Figure 1** **(A)** UMAP plots of lung T cells indicating 6 clusters: whole (left), naïve group (center) and BLM group (right). **(B)** UMAP plots indicating Foxp3 expression. **(C)** Heatmap indicating feature genes of 6 clusters. **(D)** Volcano plots showing upregulated DEGs (FC > 1.5, q-value < 0.05) in the Treg cluster versus other clusters in the BLM group (left) and the Treg cluster in the BLM group versus the naïve group (right). DEGs lists are described in **Supplementary Tables 1**, **2**. **(E)** Venn diagram of upregulated genes of indicated samples. For Bulk RNA-seq analysis, GFP^+^ CD4 T cells (Tregs) from spleens and lungs of Bleomycin-administered FDG mice were isolated. Data shows pooled samples (n = 3 biologically independent mice). Transcriptome profiles of Bulk RNA-seq are described in **Supplementary Table 3** and upregulated DEGs of lung Tregs were identified by FC > 20. **(F)** UMAP plots indicating Tff1 expression. **(G)** GFP^-^CD4 T cells (Tconv) and GFP^+^CD4 T cells (Tregs) were isolated from spleens and lungs of naïve and BLM-administered FDG mice. *Tff1* expression was measured by Q-PCR. **(H)** Tregs (Lin (B220, CD8a, CD11b, CD11c, NK1.1)^-^DAPI^-^GFP^+^) were isolated from lungs of naïve and BLM-administered FDG mice, fixed and permeabilized, and stained with DAPI and α-Tff1 antibody.

To explore immune cell status during pulmonary fibrosis unbiasedly, CD45^+^ cells from naïve and BLM-injured lungs were subjected to scRNA-seq analysis (**Figure 1** and **Supplementary Figure 1**). Datasets of Naïve lung and BLM-injured lungs post QC (**Supplementary Figure S1A**) were integrated, clustered and visualized by Uniform Manifold Approximation and Projection (UMAP) (**Supplementary Figures S1B, C**). To find Treg-specific events in pulmonary fibrosis, CD3^+^ cells-enriched clusters were applied to additional sub-clustering (**Supplementary Figure S1D** and **Figure 1A**). Among the T cell sub-clusters, cluster 5 was identified as the Treg cluster with high expression of Foxp3 (**Figures 1B, C**). In the BLM group, 267 genes were upregulated in the Treg cluster compared to other clusters, and 31 genes were upregulated in the BLM group compared to the naïve group within the Treg cluster. (**Figures 1D, E** and **Supplementary Tables 1**, **2**). Additionally, using Foxp3-reporter FDG mice (20), where GFP is expressed under the endogenous Foxp3 locus, bulk RNA-seq comparison of spleen and lung Tregs after BLM administration identified 395 genes as upregulated in lung Tregs (**Figure 1E** and **Supplementary Table 3**). Among the genes identified in these three comparisons, Tff1 remained as the only characteristic gene of BLM-lung Tregs (**Figure 1E**). In addition, Tff1 was scarcely expressed in other immune cells. (**Figure 1F** and **Supplementary Figure 1E**). To validate the *Tff1* expression found in the RNA-seq analyses, Tregs and Tconvs were collected from spleens and lungs of naïve and BLM-administered FDG mice and subjected to quantitative RT-PCR. Consistent with the RNA-seq analyses, *Tff1* expression was specifically upregulated in Tregs of BLM-injured lungs (**Figure 1G**). Also, Tff1 protein expression was observed in Tregs from BLM-injured lungs (**Figure 1H**). Collectively, these data suggest that lung Tregs specifically express Tff1 during BLM-induced pulmonary fibrosis. Therefore, we focused on Tff1-expressing Treg for further study.

### Tff1 expression in Tregs has no impact on the status of BLM-induced fibrosis

Tff1 is a kind of peptide hormone mainly secreted by gastric epithelial cells and is considered to be involved in the protection and repair of gastric mucosa (21, 22). However, less is known about the Tff1 expression in Tregs and its involvement in fibrosis. Therefore, to investigate the role of Tff1 in Tregs during pulmonary fibrosis, we generated Foxp3-Cre/*Tff1* ^fl/fl^ mice, in which Tff1 expression in Tregs was specifically disrupted (**Supplementary Figures 2A, B**). Control *Tff1* ^fl/fl^ mice and Foxp3-Cre/*Tff1* ^fl/fl^ mice were administered BLM. At 21 days post BLM administration, the degree of pulmonary fibrosis was evaluated by hydroxyproline quantification (**Supplementary Figure 2C**). However, hydroxyproline levels were comparable between control *Tff1* ^fl/fl^ mice and Foxp3-Cre/*Tff1* ^fl/fl^ mice (**Supplementary Figure 2C**). Similarly, there was no obvious difference in histopathologic inflammation and collagen deposition between the two groups (**Supplementary Figure 2D**). These data suggest that Tff1 expression itself in Tregs is not important for the condition of BLM-induced pulmonary fibrosis.

### Generation of mice targeting Tff1-expressing Tregs

**Figure 2:**
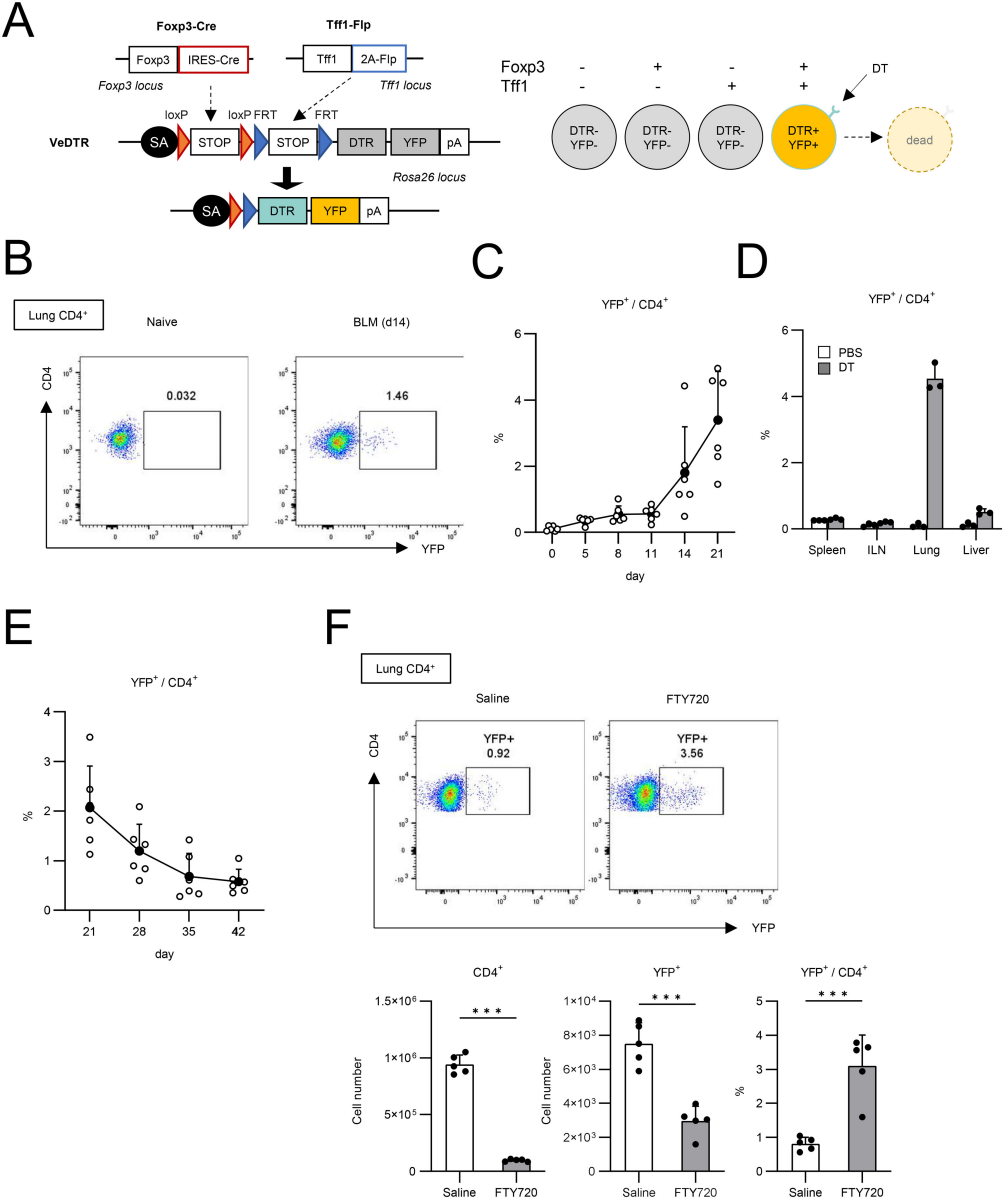
Generation of Foxp3-Cre/Tff1-Flp/VeDTR mice. **(A)** The genome editing strategy of Foxp3 and Tff1-dependent intersectional expression of YFP and DTR. **(B–F)** Foxp3-Cre/Tff1-Flp/VeDTR mice were i.t. administered with BLM. **(B, C)** Frequency of YFP^+^ in lung CD4^+^ T cells was measured over time. **(B)** Representative plot of day 0 (naïve) and day 14 post BLM administration. **(C)** Total results in indicated time points (n = 6 each). **(D)** Foxp3-Cre/Tff1-Flp/VeDTR mice were i.t. administered with PBS or BLM (n = 3 each). Frequency of YFP^+^ in CD4^+^ T cells of indicated tissues was determined on d21. **(E)** Frequency of YFP^+^ in lung CD4^+^ T cells was measured at indicated time points (n = 6 each). **(F)** Saline or FTY720 (4 µg) or daily i.p. administered in BLM-treated mice from day 0 (n=5 each). Frequency of YFP^+^ in CD4^+^ T cells of indicated tissues was determined on d14 Data means with SD. significance assessment: unpaired two-tailed Student’s t-test. ***; p<0.001.

Since we failed to find the role of Tff1 expression in Tregs, we next tried to investigate the role of Tff1-expressing Tregs (Tff1-Tregs) directly. To achieve this, we utilized the VeDTR system (19) and generated Foxp3-Cre/Tff1-Flp/VeDTR mice, in which Tff1-Tregs can be selectively labeled by YFP and depleted by DT treatment (**Figure 2A** and **Supplementary Figure S2E**). We administered BLM into the lungs of Foxp3-Cre/Tff1-Flp/VeDTR mice and measured YFP expression in lung CD4^+^ T cells (**Figure 2B**). As expected, YFP^+^ CD4^+^ T cells appeared in the lungs in response to BLM, whereas they were not detected in the lungs of naïve mice (**Figures 2B, C**). Moreover, YFP^+^ CD4^+^ T cells were preferentially localized in the lungs compared to other tissues in BLM-treated Foxp3-Cre/Tff1-Flp/VeDTR mice (**Figure 2D**). When we measured the presence of YFP^+^ CD4^+^ T cells in a time-dependent manner following the administration of BLM, percentages of YFP^+^ cells exhibited a temporal increase particularly during the transition period from the inflammatory phase to the fibrosis phase (**Figure 2C**) (23). On the other hand, following the disease peak on days 21-28 (23), YFP^+^CD4^+^ T cells began to decrease (**Figure 2E**). Taken together, these data suggest the relevance of Tff1-Tregs in the pathogenesis of BLM-induced pulmonary fibrosis.

Furthermore, we investigated whether these Tff1-Tregs were produced locally in the lung or came from the circulation (**Figure 2F**). Administration of FTY720, a T cell circulation inhibitor, reduced the number of CD4^+^ T cells in BLM-injured lungs by approximately 90%, whereas the reduction in the number of YFP^+^CD4^+^ cells was limited to around 60%, resulting in an increased proportion of YFP^+^CD4^+^ cells (**Figure 2F**). These data suggest that lung-localized Tregs are more likely to differentiate into Tff1-expressing Tregs.

### TGF-β and IL-33 induce Tff1 expression in lung Tregs

**Figure 3:**
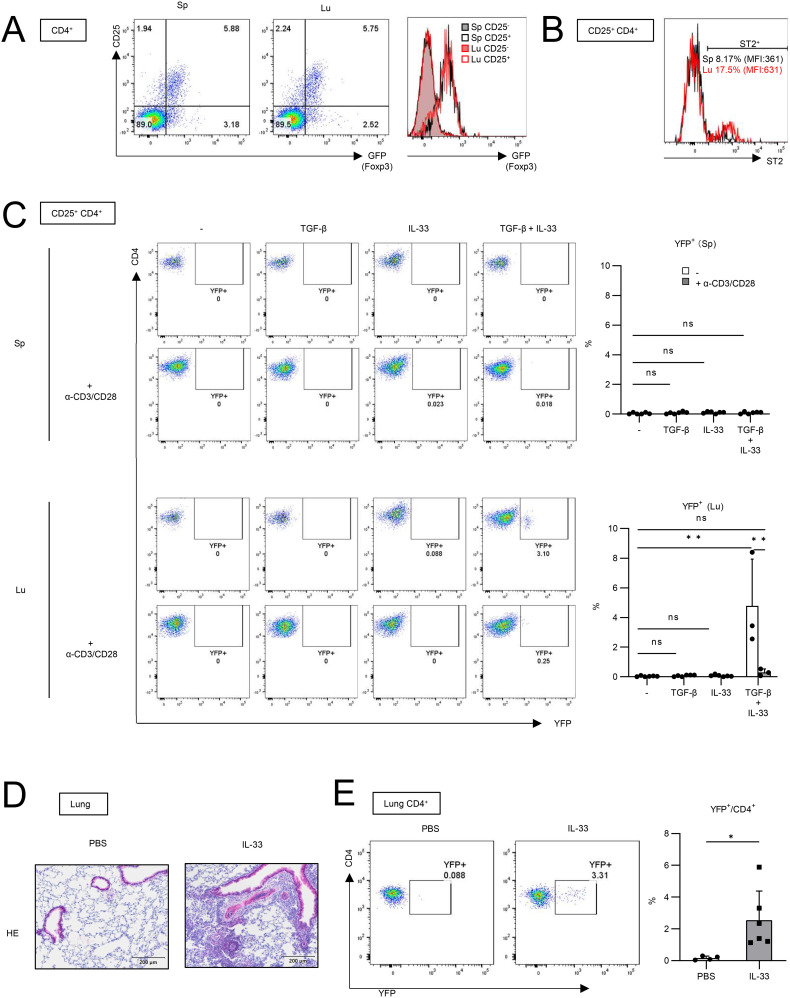
IL-33 is involved in Tff1 expression in lung Tregs *in vitro* and *in vivo*. **(A)** CD25 and GFP expression in CD4^+^ T cells from spleen (Sp) and lung (Lu) of naïve FDG mice were measured. Representative plot of two independent experiments. **(B)** ST2 expression in CD25^+^CD4^+^ T cells from spleen and lung of naïve Foxp3-Cre/Tff1-Flp/VeDTR mice was measured. Representative plot of two independent experiments. **(C)** YFP^-^CD25^+^CD4^+^ T cells from lung of naïve Foxp3-Cre/Tff1-Flp/VeDTR mice (n = 3) were cultured in the presence of indicated cytokines and α-CD3/CD28 antibody-bounded Dynabeads. 6 days later, YFP expression was measured. Representative plot (spleen: top left, lung: bottom left), and total result (spleen: top right, lung: bottom right). **(D, E)** Foxp3-Cre/Tff1-Flp/VeDTR mice were i.n. administered PBS (n = 4) or IL-33 (n = 6). **(D)** Representative image of HE staining of lung section. **(E)** Frequency of YFP^+^ in lung CD4^+^ T cells were measured Data of **(C, E)** are means with SD. significance assessment: **(C)** One-way ANOVA with a post-Tukey’s test and **(E)** unpaired two-tailed Student’s t test. **; p<0.01, *; p<0.05 and ns; not significant.

Since utilizing Foxp3-Cre/Tff1-Flp/VeDTR mice enables us to detect Tff1-Tregs by YFP expression, we sought to explore the molecular mechanism by which Tff1-Tregs can be induced *in vitro* (**Figure 3**). CD4^+^ CD25^+^ cells in naïve lymphoid tissues, such as spleen and lymph nodes, are considered as Tregs (24). To test whether CD4^+^ CD25^+^ cells in naïve lungs also can be considered as Tregs, we compared Foxp3 expression in CD25^+^ cells between splenic CD4^+^ T cells and lung CD4^+^ T cells in FDG mice (**Figure 3A**). Given that the expression of Foxp3 in lung CD4^+^ CD25^+^ cells was comparable to that in splenic CD4^+^CD25^+^ cells (**Figure 3A**), CD4^+^ CD25^+^ cells from naïve lungs were utilized as lung Tregs in subsequent analyses. In addition, lung Tregs expressed a higher level of ST2 (also known as IL-33 receptor) than splenic Tregs (**Figure 3B**), as previously reported (25, 26). In BLM-induced pulmonary fibrosis, IL-33 is expressed in the lung and promotes injury and fibrosis (27). Therefore, we investigated whether IL-33 is involved in the induction of Tff1 expression in Tregs. YFP^-^ CD4^+^ CD25^+^ cells were isolated from the spleens and lungs of naïve Foxp3-Cre/Tff1-Flp/VeDTR mice and cultured *in vitro* in the presence of IL-33 and/or TGF-β (**Figure 3C**). Stimulation of IL-33 or TGF-β alone failed to induce YFP (Tff1) expression in lung Tregs. On the other hand, it was noteworthy that the combination of both IL-33 and TGF-β successfully induced YFP expression in lung Tregs, but not in splenic Tregs (**Figure 3C**). Unexpectedly, α-CD3/CD28 stimulation did not promote Tff1-Treg induction by IL-33 and TGF-β, but rather inhibited it (**Figure 3C**).

Next, we investigated whether IL-33 can induce Tff1-Tregs *in vivo.* Intranasal administration of IL-33 has been shown to induce pulmonary inflammation including the accumulation of eosinophils, resulting in asthma (28, 29). IL-33 was also reported to induce TGF-β expression from eosinophils or lung epithelium (30, 31). When we intranasally administered IL-33 into Foxp3-Cre/Tff1-Flp/VeDTR mice, the IL-33 treatment successfully induced pulmonary inflammation (**Figure 3D**). Then, we found that YFP^+^ CD4^+^ cells significantly increased in the IL-33-administered lung (**Figure 3E**).

Since TGF-β and IL-33 appeared to be important factors for inducing Tff1-Tregs, we measured their expression in the BLM-treated lungs (**Supplementary Figure 3A**). TGF-β expression closely resembled the kinetics of Tff1-Treg increase, showing a gradual increase over time (**Figure 2C** and **Supplementary Figure S3A**). On the other hand, IL-33 exhibited transient high expression at an earlier time (**Supplementary Figure S3A**). Furthermore, to determine whether TGF-β and IL-33 are essential for the induction of Tff1-Tregs *in vivo*, we examined YFP expression during BLM treatment with IL-33 and TGF-β neutralizing antibodies. α-IL-33 antibody alone did not change the frequency of YFP^+^ cells. In contrast, α-TGF-β antibody reduced the frequency of Tff1^+^ Tregs. The effect was comparable between α-TGF-β antibody alone and the combination with α-IL-33 (**Supplementary Figure 3B**). These data suggest that TGF-β and IL-33 are essential and dispensable for inducing Tff1-Tregs *in vivo*, respectively.

### Depletion of Tff1-Tregs exacerbates pulmonary fibrosis

**Figure 4:**
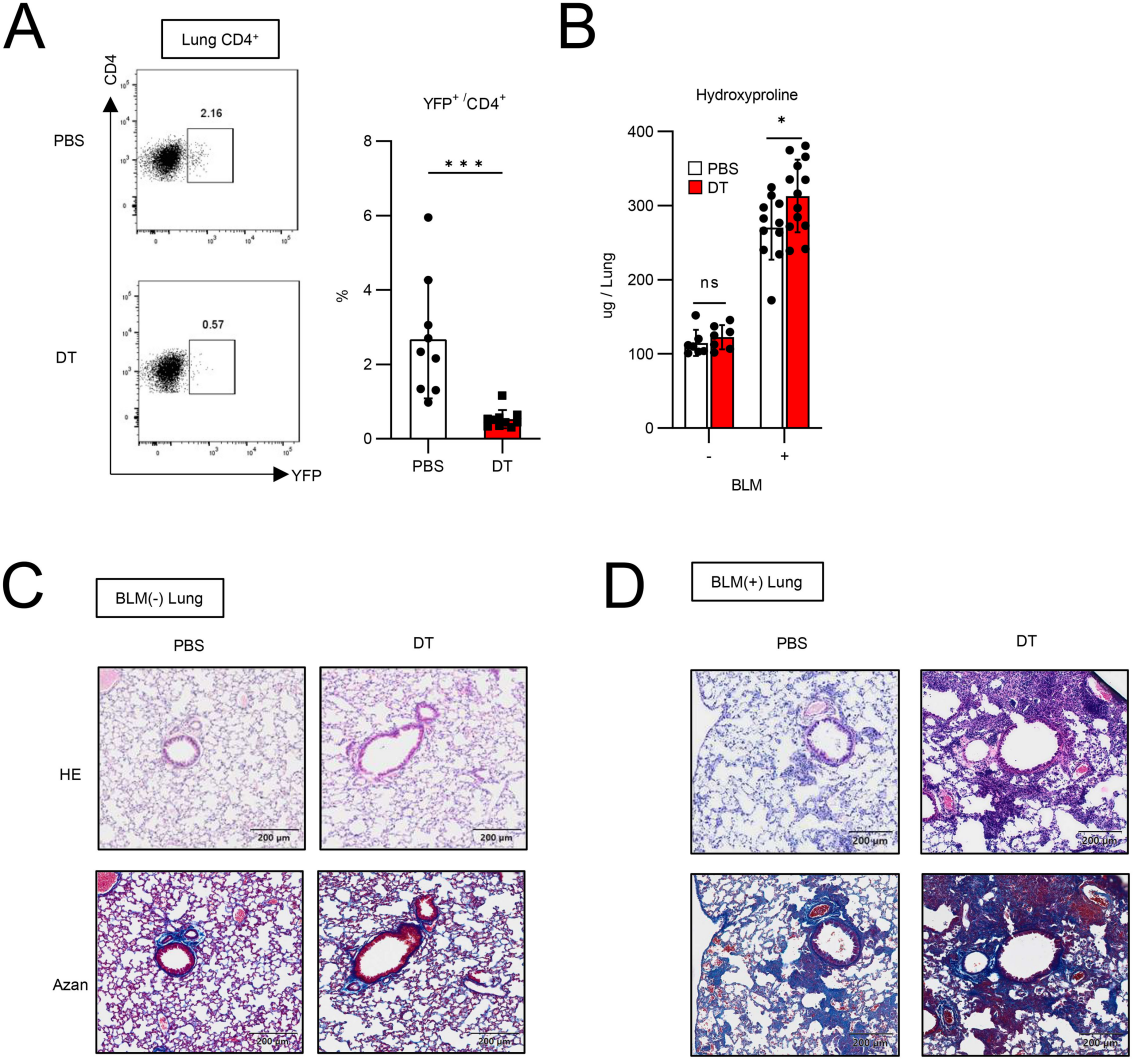
Ablation of Tff1-Tregs progress fibrosis. **(A–D)** Foxp3-Cre/Tbff1-Flp/VeDTR mice were administered or not administered BLM. PBS or DT (100ng) was i.p. administered every 3 days from day 7. Each analysis was conducted on day 21. **(A)** The frequency of YFP^+^ cells in lung CD4^+^ T cells was measured by flow cytometry. Representative plot (left) and total results (right) (PBS: n = 9, DT: n = 10). **(B)** Hydroxyproline levels in lung were measured. (BLM(-) PBS: n = 7 BLM(-) DT: n = 7, BLM(+) PBS: n = 11, BLM(+) DT: n = 13). **(C)** HE and Azan staining of lung section of BLM-non-administered mice and **(D)** BLM-administered mice. Data of **(A, B)** are means with SD. significance assessment: unpaired two-tailed Student’s t test. ***; p<0.001, *; p<0.05 and ns; not significant.

Although Tff1-Tregs appeared in inflamed lungs (**Figures 1** **–** **3**), how these cell types impact the pathology remained unclear. To address this question, we assessed the effect of Tff1-Treg depletion on the severity of pulmonary fibrosis. DT treatment in BLM-administered Foxp3-Cre/Tff1-Flp/VeDTR mice efficiently depleted YFP^+^ cells in the lungs (**Figure 4A**). When evaluating the effect of Tff1-Treg depletion by DT, naïve lungs did not exhibit a change in hydroxyproline levels or histopathology by DT treatment (**Figures 4B, C**). Notably, DT treatment in BLM-administered mice showed increased hydroxyproline levels and severe histopathological inflammation and collagen deposition in the lungs (**Figures 4B, D**). Taken together, these results indicate that Tff1-Tregs play a protective role in preventing the exacerbation of BLM-induced pulmonary fibrosis.

### Tff1-Tregs depletion increases neutrophils and inflammatory myeloid cells

**Figure 5:**
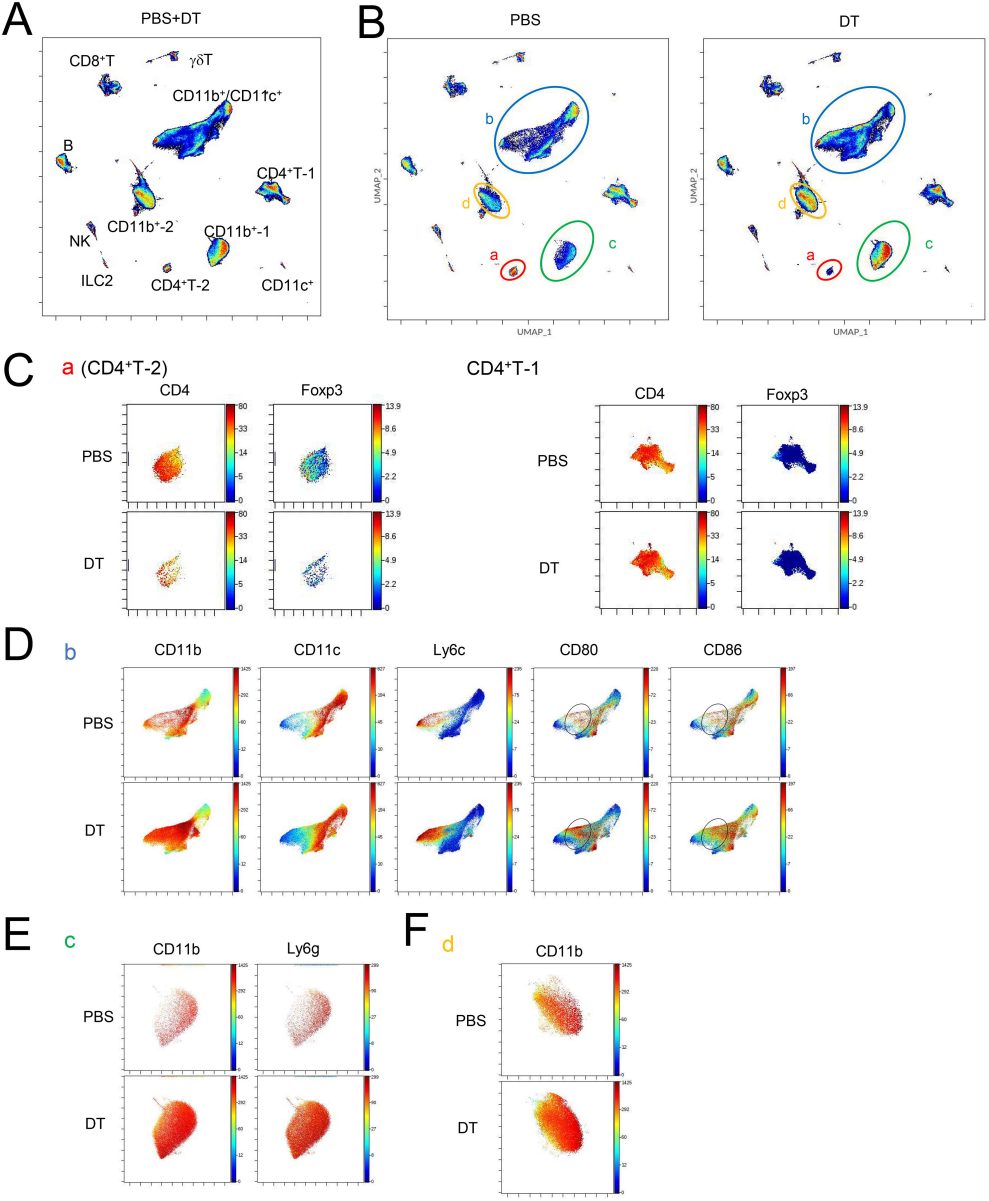
Ablation of Tff1-Tregs changes immune landscape. **(A–D)** Foxp3-Cre/Tbff1-Flp/VeDTR mice were administered BLM. PBS or DT (100ng) was i.p. administered every 3 days from day 7 (n = 3 each). Lung CD45^+^ cells were subjected to CyTOF analysis on day 21. UMAP-plot **(A)** of whole condition and **(B)** of PBS (left) and DT condition (right). Expression of indicated genes in **(C)** population “a” and CD4^+^ T-1, **(D)** “b” **(E)** “c” and **(F)** “d”, which are annotated in **(B)**. See also **Supplementary Figure 3**.

Next, we explored the mechanism by which Tff1-Tregs suppress exacerbation of BLM-induced pulmonary fibrotic conditions. To unbiasedly search for changes in the landscapes of multiple immune cell populations in the lungs induced by the depletion of Tff1-Tregs, we next employed cytometry by time-of-flight (CyTOF). The landscape of lung CD45^+^ cells of PBS- and DT-treated BLM-administered Foxp3-Cre/Tff1-Flp/VeDTR mice was visualized by UMAP and annotated each aggregated population by immune cell markers (**Figure 5A** and **Supplementary Figure 4**). Among them, when comparing the DT group to the PBS group, four distinct populations clearly decreased (a: CD4^+^T-2) or increased (b: CD11b^+^/CD11c^+^, c: CD11b^+^-1, d: CD11b^+^-2) in the DT group (**Figure 5B**). The decreased population “a” was CD4^+^ Foxp3^+^ T cells, which appears to reflect the depletion of Tff1-Tregs by DT treatment, while another CD4^+^ population (CD4^+^ T-1) was likely to be Foxp3-negative Tconvs (**Figure 5C**). Of the increased populations, population “b” was a group of cells with varying expressions of CD11b, CD11c, and Ly6C, indicating that they were likely composed of a mixed population of myeloid cells including monocytes and macrophages such as alveolar macrophages (AMs) (**Figure 5D**). In particular, CD80 and CD86 highly expressing cells were increased in the population “b” of the DT group (**Figure 5D**), suggesting that Tff1-Treg depletion might enhance the inflammatory phenotype of these cells. Given that CD11b and Ly6G were highly expressed, the population “c” was likely neutrophils (**Figure 5E**). Regarding population “d”, we could not characterize them beyond their identification as CD11b^+^ cells with our panel design (**Figure 5F** and **Supplementary Figure 4**).

**Figure 6:**
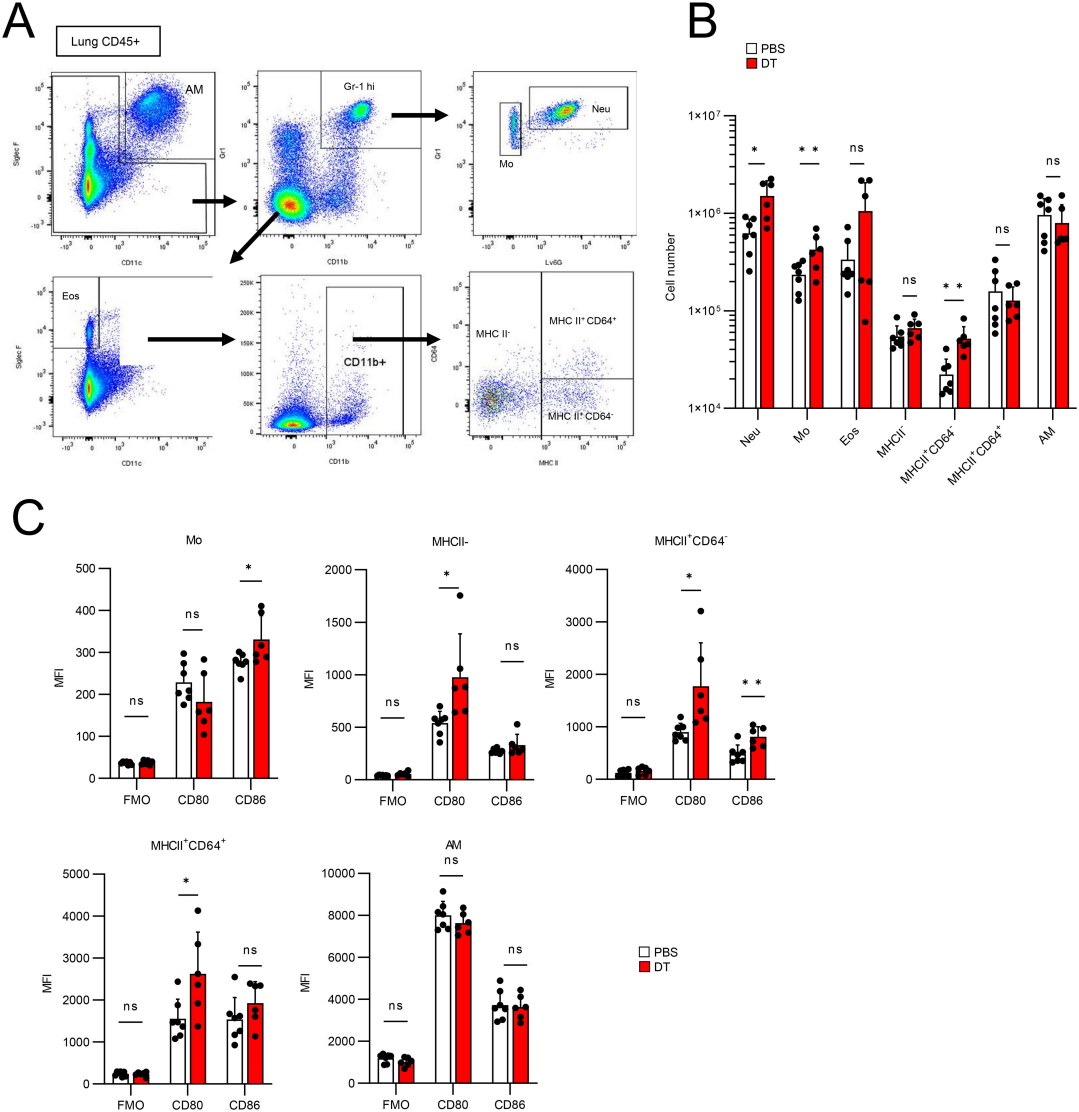
Ablation of Tff1-Tregs enhance proinflammatory features of myeloid cells. **(A–C)** Foxp3-Cre/Tbff1-Flp/VeDTR mice were administered BLM. PBS (n = 7) or DT (100ng) (n = 6)was i.p. administered every 3 days from day 7. Lung CD45^+^ cells were subjected to flow cytometry analysis on day 21. **(A)** Gating strategy for discriminating the indicated cell populations. **(B)** Cell number of each population. **(C)** Expression level of the CD80 and CD86 in indicated population. Data of **(B, C)** are means with SD. Statistical significance assessment: unpaired two-tailed Student’s t test. **; p<0.01, *; p<0.05 and ns; not significant.

Next, we confirmed the validity of the results obtained from CyTOF using flow cytometry (**Figure 6**). We classified lung CD45^+^ cells into neutrophils (Gr1^hi^ Ly6G^+^ CD11b^+^), monocytes (Gr1^hi^ Ly6G^-^ CD11b^+^), eosinophils (SiglecF^+^ CD11c^-^), MHCII^-^ CD11b^+^ cells, MHCII^+^ CD64^-^ CD11b^+^ cells, MHCII^+^ CD64^+^ CD11b^+^ cells, which are typical interstitial macrophages (IMs), and AMs (SiglecF^+^ CD11c^+^) (**Figure 6A**), and compared their cell numbers in PBS- or DT-treated BLM-administered Foxp3-Cre/Tff1-Flp/VeDTR mice (**Figure 6B**). The numbers of neutrophils, monocytes and MHCII^+^ CD64^-^ CD11b^+^ cells significantly increased in DT-treated mice (**Figure 6B**). In addition, when comparing CD80 and CD86 expression, CD80 expression was significantly increased in MHCII^-^ CD11b^+^ cells, MHCII^+^ CD64^-^ CD11b^+^ cells and IMs from the DT-treated mice compared to those from the PBS-treated mice (**Figure 6C**). CD86 expression was also markedly increased in monocytes and MHCII^+^ CD64^-^ CD11b^+^ cells from the DT-treated mice (**Figure 6C**), indicating that depletion of Tff1-Tregs enhances inflammatory phenotypes of macrophages and monocytes in the inflamed lungs. Collectively, these data suggest that Tff1-Tregs may suppress the accumulation of neutrophils and the inflammatory state of macrophages and monocytes to prevent the exacerbation of fibrosis.

## Discussion

In this study, we revealed a unique subset of Tregs expressing Tff1, which are not present in healthy lungs but emerge during BLM-induced pulmonary fibrosis, contributing to the inhibition of disease progression. Recently, Li et al. have shown that aorta Tregs also express Tff1 in elastase- or calcium phosphate-induced abdominal aortic aneurysms (AAA) (32). Additionally, their creation of Foxp3-Cre/*Tff1* ^fl/fl^ mice demonstrated that Treg-derived Tff1 contributes to promoting tissue repair. However, unlike in AAA, our examination using Foxp3-Cre/*Tff1* ^fl/fl^ mice in the BLM-induced pulmonary fibrosis model revealed that the absence of Tff1 produced by Tregs did not affect the degree of fibrosis. Thus, although the role of Tff1 in BLM-induced pulmonary fibrosis remains unclear, Tff1 itself may play a limited role.

Our study highlights the cellular significance of Tff1-Tregs rather than the molecular role of Treg-derived Tff1. Regarding the induction of Tff1 in Tregs, we found the involvement of IL-33 and TGF-β. Interestingly, *in vitro* experiments demonstrated that Tff1-Tregs were not induced under α-CD3/CD28 antibody stimulation even in the presence of IL-33 and TGF-β. This suggests that Tff1-Tregs might represent a response of bystander-activated Tregs rather than being driven by antigen-specific TCR-mediated activation (33). Furthermore, Tff1-Tregs were observed in only a limited proportion of cultured lung Tregs, suggesting that full induction of Tff1-Tregs might require additional stimuli, or that there exists a subpopulation of lung Tregs capable of expressing Tff1. Alternatively, Tff1 expression might simply dependent on the strength of signaling proportional to ST2 expression. This is because, although there is also an ST2^+^ population in splenic Tregs, they have lower ST2 levels than lung Tregs. Also, splenic Tregs failed to express Tff1 in the presence of IL-33 and TGF-β. On the other hand, the neutralization of IL-33 did not affect the induction of Tff1-Tregs *in vivo*. For IL-33, other cytokines, such as other IL-1 family molecules to which IL-33 belongs, may compensate for its function *in vivo*. Future research investigating the relationship between TCR stimulation and Tff1-Treg induction *in vivo*, as well as identifying factors that govern the potential for Tff1-Treg differentiation would be interesting.

Depletion of Tff1-Tregs exacerbated the fibrotic state of BLM-injured lungs, indicating that Tff1-Tregs contribute to suppressing fibrosis progression. Although both IL-33 and TGF-β are considered as pro-fibrotic cytokines (11, 12, 27), the induction of Tff1-Tregs by these cytokines might be part of a feedback mechanism for maintaining biological homeostasis. In the absence of Tff1-Tregs, the numbers of neutrophils, macrophages and monocytes were increased at the pathogenic site. In addition, the expression of CD80 and CD86, typical markers for an inflammatory phenotype of myeloid cells (34), on the macrophages and monocytes also increased in the absence of Tff1-Tregs. Sustained inflammatory response has been shown to enhance pulmonary fibrosis (35, 36). Collectively, our data, together with these previous findings, suggest that Tff1-Tregs may ameliorate lung inflammation during fibrosis by decreasing the number and inactivating these myeloid cells.

Currently, it is commonly understood that Tregs are a population composed of very heterogeneous communities. Considering multiple studies, the effect of removal or adoptive transfer of Tregs on BLM-induced fibrosis has different results, either reducing or exacerbating fibrosis, depending on the methodology and timing of intervention (16–18, 37, 38). These variations suggest dynamic changes in the Treg community before and after BLM treatment. Consequently, it is likely inappropriate to define the function of Tregs as a monolith. In this study, we demonstrated that Tff1-Tregs, emerging as a part of diverse Treg populations post-BLM administration, contribute to the suppression of fibrosis progression. Understanding the heterogeneity of Tregs in fibrosis might provide insight into novel therapeutic approaches to treating human IPF.

## Methods

### Mice

C57BL/6N mice were purchased from Japan SLC., Foxp3-Cre mice, VeDTR mice and FDG mice were described as previously. All animal experiments were approved by the Animal Research Committee of Research Institute for Microbial Diseases at Osaka University.

### Reagents

DT was purchased from Millipore. FTY720 was purchased from Sigma-Aldrich. Antibodies used in this study are described in **Supplementary Table S4**.

### Generation of *Tff1* ^fl/fl^ mice and Tff1-flp mice by genome editing

The T7 promoter sequence-containing gRNA1, gRNA2 and gRNA3 PCR products were amplified by the primers (Tff1flox_gRNA1_F (5’- TTAATACGACTCACTATAGGgcttgccgcatctacagatgGTTTTAGAGCTAGAAATAGCAAGTTAAAAT -3’) and gRNA_common_R2 (5’- AAAAGCACCGACTCGGTGCCACTTTTTCAAGTTGATAACGGACTAGCCTTATTTTAACTTGCTATTTCTAGCTCT -3’) for gRNA1; Tff1flox_gRNA2_F (5’- TTAATACGACTCACTATAGGcctgtgacatagctgaatccGTTTTAGAGCTAGAAATAGCAAGTTAAAAT -3’) and gRNA_common_R2 for gRNA2; Tff1KI_gRNA3_F (5’- TTAATACGACTCACTATAGGttagaagaatgtcccttctaGTTTTAGAGCTAGAAATAGCAAGTTAAAAT -3’) and gRNA_common_R2 for gRNA3) using KOD FX NEO (Toyobo), respectively. MEGAshortscript T7 (Life Technologies) was used for the generation of these gRNAs using each of the gRNA PCR products. Cas9 mRNA was generated by *in vitro* transcription (IVT) using mMESSAGE mMACHINE T7 ULTRA kit (Life technologies) and the template that was amplified by PCR using pEF6-hCas9-Puro and the primers T7Cas9_IVT_F and Cas9_R (Okamoto et al. Cell Rep. 2023), and gel-purified. The synthesized gRNA and Cas9 mRNA were purified using MEGAclear kit (Life Technologies). For generation of the targeting fragment for the floxed *Tff1* allele, the *Tff1* gene was isolated from genomic DNA that was extracted from C57BL/6N embryonic fibroblasts by PCR using KOD FX NEO (Toyobo) and primers (Tff1_flox_LA_F (5’- gtcgacGTGACCCCTTATTGTCCTGTGCTGTGGCCAGTGCTCATCTTAGCTTTATATTAG -3’) and Tff1_flox_LA_R (5’- agatcttgtagatgcggcaagcCTGGCGCTGGGACAGTTCCAGGGCCACCGCTTCCTCAG -3’) for the LA fragment; Tff1_flox_MA_F (5’- agatctATAACTTCGTATAGCATACATTATACGAAGTTATgatgAGGACTGTATCCCCTTGCATATTCAAACCTGGCTTC -3’) and Tff1_flox_MA_R (5’- acgcgttcagctatgtcacaggAATCCAGGGACAGGGAAAGACCAACCAGAGATACGGAC -3’) for the MA fragment; Tff1_flox_RA (5’- acgcgtATAACTTCGTATAGCATACATTATACGAAGTTATatccAGGTCTTGGGGGGTGAAGGGTCAAGGCTAAAAGTCT -3’) and Tff1_flox_RA_R (5’- gcggccgcGGTTTGGTTCCCAGCACCAGCTAATTCTTTTAAAGGGCATCAAATTCCATAA -3’) for the RA fragment). The MA fragment contains exons 2. The LA, MA and RA fragments were ligated using restriction enzymes in pBluescript. The vectors were amplified and co-injected into the embryos with the Cas9-encoding mRNA, gRNA1 and gRNA2 to obtain *Tff1* ^fl/+^ pups. *Tff1* ^fl/+^ mice were further crossed with Foxp3-Cre mice to generate Foxp3-Cre *Tff1* ^fl/fl^ mice.

For the generation of the targeting fragment for the *Tff1* allele C-terminally fused with P2A-sequence and optimized Flp recombinase (P2A-Flp) cassette (Okamoto et al. Cell Rep. 2023), the *Tff1* gene was isolated from genomic DNA that was extracted from C57BL/6N embryonic fibroblasts by PCR using KOD FX NEO (Toyobo) and primers (Tff1_KI_LA_F (5’- gaattcATGTGTGAGGCCAGATGTCAACACCATGTCTCATCACTCAGGAGCCACTCTTT -3’) and Tff1_KI_LA_R (5’- ctcgagGAAGGGACATTCTTCTAAAGAGAGAAGAACAAAGGGTGAGAGACCAGACAAACT -3’) for the LA fragment; Tff1_KI_RA_F (5’- ggatccGGTCCATCCTGAGAGAACTGGCTACATCAAGACTTGGCACCCTCCACCTGGGCA -3’) and Tff1_KI_RA_R (5’- gcggccgcGACAAGGCGATGGATAGAACCATGCATTGACCAGACTGCTTAGCTGTGCATA -3’) for the RA fragment). The LA, Flp and RA fragments were ligated using restriction enzymes in pBluescript. The vectors were amplified and co-injected into the embryos with the Cas9-encoding mRNA, gRNA3 to obtain pups for Tff1-Flp mice. Tff1-Flp mice were further crossed with Foxp3-Cre mice and VeDTR mice to generate Foxp3-Cre/Tff1-Flp/VeDTR mice.

### Bleomycin-induced pulmonary fibrosis

10-16 weeks old Age- and sex-matched mice were anesthetized with a mixture of medetomidine-midazolam-butorphanol and intratracheally administered with 50 μg Bleomycin (Nippon Kayaku) in 50 μl PBS.

### Cell preparation from mice tissues

To prepare cells from lung, tissue was minced to 2-3 mm with scissors in 2 ml HBSS containing 1% FCS, followed by Liberase TM (50 μg/m) and DNase I (50 μg/ml) were added, incubated for 1 hrs at 37 °C with shaking (160 rpm), then 7 ml HBSS was added and dissociated using MACS Octo Dissociator (Miltenyi), employing preset programs C. After dissociation, the cells were passed thorough 100 μm filter and centrifuged for 5 min at 2000 rpm. The cell pellet was resuspended in ACK buffer and incubated for 2 minutes at room temperature and then washed with HBSS. The washed cells were resuspended in HBSS with 40% Percoll (Sigma-Aldrich) and centrifuged for 20 min at 2,380×g to eliminate floating debris. Finally, the resulting pellet was rinsed with HBSS. Prepared cells were resuspended in the appropriate buffer for each experiment. Cell preparation from other tissues was described previously.

### Flow cytometry and cell sorting

Cells were first blocked with anti-CD16/32 antibody in 2% BSA in PBS for 5 min on ice. For surface staining, cells were stained with antibodies in 2% BSA in PBS for 15 min on ice and then washed twice in 2% BSA in PBS. For live/dead cell discrimination, DAPI (NacaraiTesque), 7AAD (BD Pharmingen) or Fixable Viability Dye eFluor 450 (Invitrogen) were used. FACSymphony A1 (BD) or FACS Aria III (BD) was used for data acquisition or cell sorting. Precision Count Beads (BioLegend) was used to obtain cell numbers. The acquired data were analyzed using FlowJo ver. 10.8.0 (BD).

### Single-cell RNA seq data acquisition

Living CD45^+^ cells were sorted from naïve lung or BLM-injured lung (day 15) of WT mice. The sorted samples from three individual mice under identical conditions were pooled together, ensuring an equal cell count across the combined sample. Single-cell RNA-seq analysis was conducted using BD Rhapsody Single-Cell Analysis System (BD BioScience) following the manufacturer’s protocol. For library construction, we used BD Rhapsody Whole transcriptome analysis (WTA) Amplification Kit. Sequencing was performed on HiSeq 3000 platform in a 100 + 100 base paired-end mode. Generated reads were input into the BD Rhapsody WTA Analysis Pipeline for UMI counting for each cell(index)-gene combination.

### Single-cell RNA seq data analysis

The processed data obtained from BD Rhapsody WTA Analysis Pipeline were loaded into SeqGeq software ver 1.6 (BD) and performed the quality control following the instruction (https://docs.flowjo.com/seqgeq/quality-control/). Subsequently, Seurat was used for further analysis.

### Bulk RNA-seq analysis

For Bulk RNA-seq Analysis, GFP^+^CD4^+^ T cells were sorted from lung and spleen of BLM-administered FDG-mice at day 15. Total RNA was extracted from cells with TRIzol reagent and a miRNeasy Micro kit (Qiagen) following the manufacturer’s instruction. Full-length cDNA was prepared using a SMART-Seq HT Kit (Takara Bio, Japan). According to the SMART-Seq kit instructions, an Illumina library was then prepared using a NexteraXT DNA Library Preparation Kit (Illumina). Sequencing was performed on HiSeq 2500 platform in a 75-base single-end mode. Generated reads were mapped to the mouse (mm10) reference genome using TopHat v2.1.1 in combination with Bowtie2 ver. 2.2.8 and SAMtools ver. 0.1.18. Fragments per kilobase of exon per million mapped fragments (FPKMs) were calculated using Cuffdiff 2.2.1.

### Quantitative PCR

For quantitative PCR, total RNA was extracted using RNeasy Mini Kit (Qiagen), after which RNA was reverse transcribed using Verso cDNA synthesis Kit (Thermo Scientific) following the manufacturer’s instructions. Quantitative PCR was performed using GoTaq qPCR Master Mix (Promega) and CFX Connect (Bio-Rad). The expression of mRNA was normalized to that of *Actb* mRNA. Primer sets used for amplification were as follows: mouse *Tff1* forward: 5′- AGCACAAGGTGATCTGTGTCC-3′ and reverse: 5′-GGGGGCCATGATACATGTTTC-3′; mouse *Actb* forward: 5′-TTTGCAGCTCCTTCGTTGC-3′ and reverse: 5′-TCGTCATCCATGGCGAACT-3′; mouse Tgfb forward: 5′-GGAGAGCCCTGGATACCAACTAT-3′ and reverse: 5′- CCAGACAGAAGTTGGCATGGT-3′; mouse Il33 forward: 5′-AAGACCAGGTGCTACTACGC-3′ and reverse: 5′-CTTCTTCCCATCCACACCGT-3′.

### Induction of Tff1-expressing Treg *in vitro*

YFP^-^ Treg cells (Lin (B220, CD8a, CD11b, CD11c, NK1.1)^-^CD4^+^CD25^+^ YFP^-^) were sorted from spleens and lungs of Foxp3-Cre/Tff1-Flp/VeDTR mice. 3000-5000 cells were cultured in 96-well U-bottom plates with RPMI1640 (Nacalai Tesque) containing 10% heat-inactivated FCS (Gibco), 100 U/ml Penicillin/Streptomycin (Nacalai Tesque), 50 mM 2-ME (Nacalai Tesque), and 20 ng/ml mIL-2 (Pepro Tech). Cultures were additionally supplemented with 5 ng/ml hTGF-β (Pepro Tech) and/or 50 ng/ml mIL-33 (R&D biosystems). For CD3/CD28 stimulation, Dynabeads Mouse T-Activator CD3/CD28 (Gibco) in equal numbers of cells were added to the cultures. Cells were cultured for 6 days and then analyzed.

### Hydroxyproline assay

For sample preparation, lung tissue was homogenized in water using a PT1300D (KINEMATICA), an equal volume of 12N HCl to the homogenate was added, incubated for 3 hrs at 120°C, and then diluted fivefold with water. For quantification, to each 25 µl the sample or hydroxyproline standards, 100 µl oxidant reagent (7% chloramine T: citric acid buffer (pH 6.0, 5.7% w/v sodium acetate trihydrate, 3.75% w/v trisodium citrate dihydrate, 0.55% w/v citric acid monohydrate, and 38.5% v/v isopropanol) = 1:4) was added, and the mixture was incubated for 20 min at room temperature. Subsequently, 100 µl Ehrlich’s reagent (p-DMAB: 60% perchloric acid: isopropanol = 3 (w): 5 (v): 10 (v)) was added, and the mixture was further incubated for 20 min at 65°C. Finally, the optical density at 560 nm was measured. All chemicals were purchased from Nakarai Tesque.

### Immunofluorescence assay

For immunofluorescence assay, Tregs from the lungs of FDG mice were transferred on a glass plate by cytospin and fixed in cold acetone for 10 min at -20°C. After fixation, they were incubated in Blocking Buffer (PBS with 0.1% BSA, 1% mouse serum, 1% donkey serum and 1% goat serum) for 1 hr at RT. They were then primarily stained with the antibodies in Blocking Buffer overnight at 4°C and washed three times with PBS. The sections were subsequently stained with secondary antibody for 1hr RT washed three times. The stained sections were mounted using ProLong Diamond Antifade Mountant (Invitrogen). The images were observed using FV3000 (Olympus).

### Histopathological staining

For histopathological staining, lungs were fixed by degassing in 10% formalin and post-fixed for 72-96 hrs at 4°C. The fixed tissues were embedded in paraffin and sectioned at 5 µm using REM-710 (Yamato). Sections were applied to HE staining or Azan staining, and mounted with Mount-Quick (Daido Sangyo). The images were observed using VS200 (Evident).

### CyTOF analysis

The staining procedure and data acquisition were previously described (19). Briefly, each sample was first barcoded by different metal-conjugated α-CD45 antibodies, pooled, and subsequential staining and data acquisition by Helios (Standard BioTools) were performed in a single tube. Antibodies used for mass cytometry are listed in **Supplementary Table 5**. For acquired data analysis, live singlet cell gating and sample debarcoding were performed using FlowJo and subsequential analysis was performed using Cytobank ver. 10.3 (Beckman Coulter). UMAP algorithm with proportional sampling in Cytobank was used for 2D mapping.

### Statistical analysis

For Statistical significance assessment, an unpaired two-tailed Student’s t-test was performed to compare the two groups. One-way ANOVA with a post-Tukey’s test was performed to compare multiple groups. One-way ANOVA with a post-Dunnett’s test was performed for many-to-one comparison. Statistical significance values are indicated as follows: ns = not significant, ∗ = p < 0.05, ∗∗ = p < 0.01, and ∗∗∗ = p < 0.001.

## Data Availability

The datasets presented in this study can be found in online repositories. The names of the repository/repositories and accession number(s) can be found below: GSE267863 and GSE267844 (GEO).

## Supplementary material

Supplementary Figure 1 Additional information of scRNA-seq analysis in **Figure 1** **(A)** QC metrics for scRNA-seq datasets. Cells were filtered based on library size, number of expressed genes, and mitochondrial DNA content percentage. **(B)** UMAP plots of lung CD45+ cells indicating 19 clusters: whole (top), naïve group (bottom left) and BLM group (bottom right). **(C)** Heatmap indicating feature genes of 19 clusters. **(D)** UMAP plots indicating Cd3e expression. Based on Cd3e expression, clusters 4,8,9 and 11 were sub-cluster and further analyzed in **Figures 1A–F** **(E)** UMAP plots indicating Tff1 expression.

Supplementary Figure 2 Tff1 disruption in Tregs has less impact on extend of fibrosis **(A)** Targeting strategy for the floxed *Tff1* locus. The exon 2 was flanked by loxP sequences. **(B)** genomic PCR for Tff1 and actb genes was performed on indicated cells **(C)** Ctrl *Tff1* ^fl/fl^ mice and Foxp3-Cre/*Tff1* ^fl/fl^ mice were administered BLM. Hydroxyproline levels in lung were measured on day21. (Ctrl *Tff1* ^fl/fl^: n = 11, Foxp3-Cre/*Tff1* ^fl/fl^: n = 13). Data are means with SD. Statistical significance assessment: unpaired two-tailed Student’s t test. ns; not significant. **(D)** HE and Azan staining of lung section of BLM-administered Ctrl *Tff1* ^fl/fl^ and Foxp3-Cre/*Tff1* ^fl/fl^ mice. **(E)** Targeting strategy for the *Tff1* locus to insert the P2A-Flp cassette.

Supplementary Figure 3 TGF-β is essential for inducing Tff1-Treg *in vivo* **(A)** WT mice were i.t. administered with BLM. *Tgfb* and *Il33* mRNA expression was measured by Q-PCR at indicated time points (n = 4 each). **(B)** Foxp3-Cre/Tff1-Flp/VeDTR mice were i.t. administered with BLM. In addition, α-TGF-β (200 µg) and α-IL-33 (4 µg) antibodies, both individually and in combination, or isotype ctrl mix (200 µg mouse IgG1 and 4 µg goat IgG) i.p. administered on day 0, 4, 8 and 12. Frequency of YFP^+^ in CD4^+^ T cells of indicated tissues was determined on day 14. Data are means with SD. significance assessment: One-way ANOVA with a post-Tukey’s test. **; p<0.01, *; p<0.05 and ns; not significant.

Supplementary Figure 4 Heatmaps of each channel in CyTOF analysis Heatmaps indicating the expression level of each channel in entire population in CyTOF analysis. Related to **Figure 5**. The molecules written in red were used for cell classification in **Figure 5A**.

Supplementary Table 1 Upregulated genes of Tregs in BLM-injured lung vs rest in BLM-injured lung (scRNA-seq).

Supplementary Table 2 Upregulated genes of Tregs in BLM-injured lung vs Tregs in naïve lung (scRNA-seq).

Supplementary Table 3 Transcriptional profile of lung and splenic Tregs of BLM-administered mice (Bulk-RNA seq).

Supplementary Table 4 List of antibodies.

Supplementary Table 5 List of antibodies for CyTOF.
