# Supplementary material for: Tff1-expressing Tregs in lung prevent exacerbation of Bleomycin-induced pulmonary fibrosis
Source: Front Immunol. 2024 Sep 2;15:1440918. doi: 10.3389/fimmu.2024.1440918 (PMC11402662; doi:10.3389/fimmu.2024.1440918)

Supplementary Figure 1

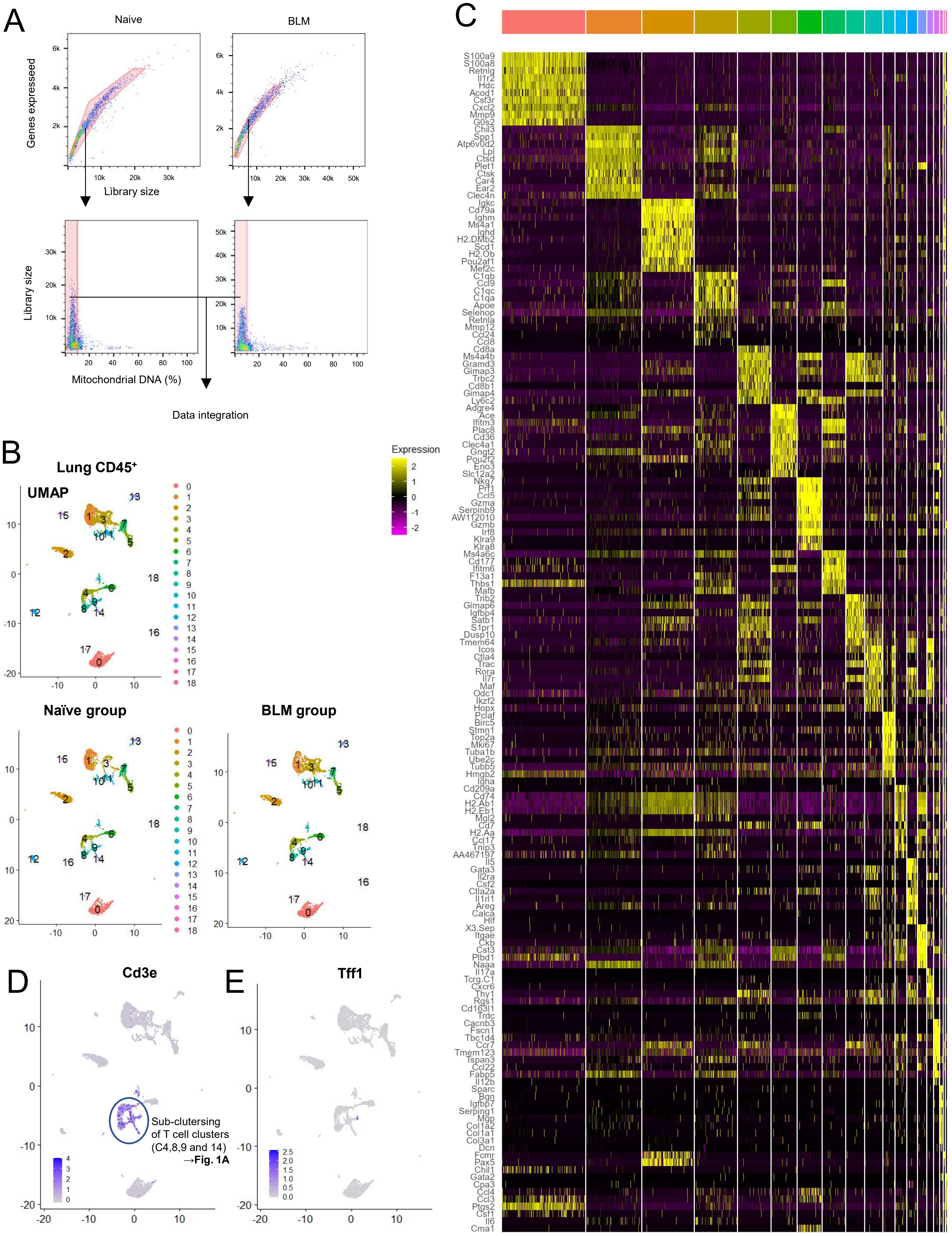

Supplementary Figure 2

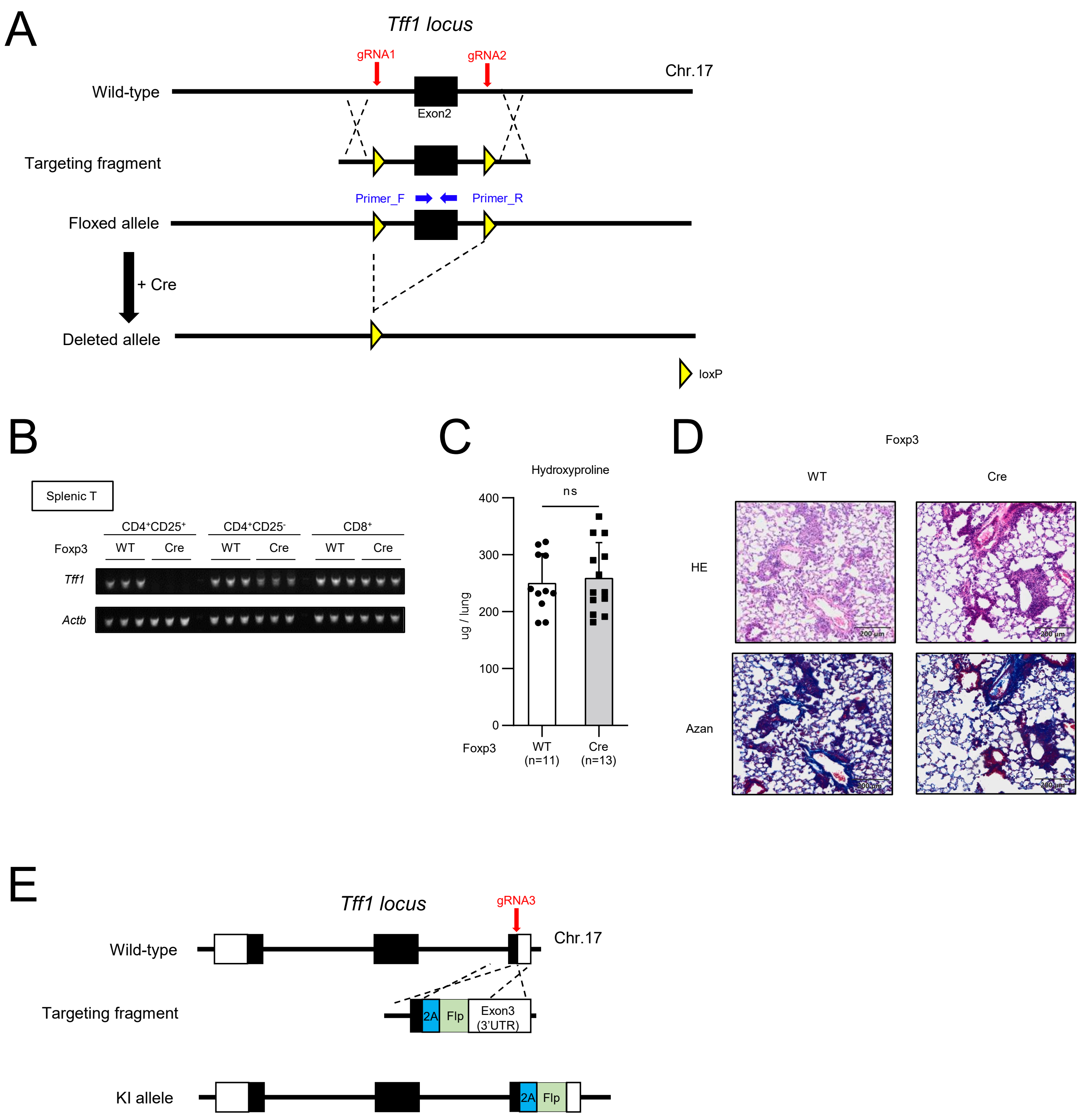

Supplementary Figure 3

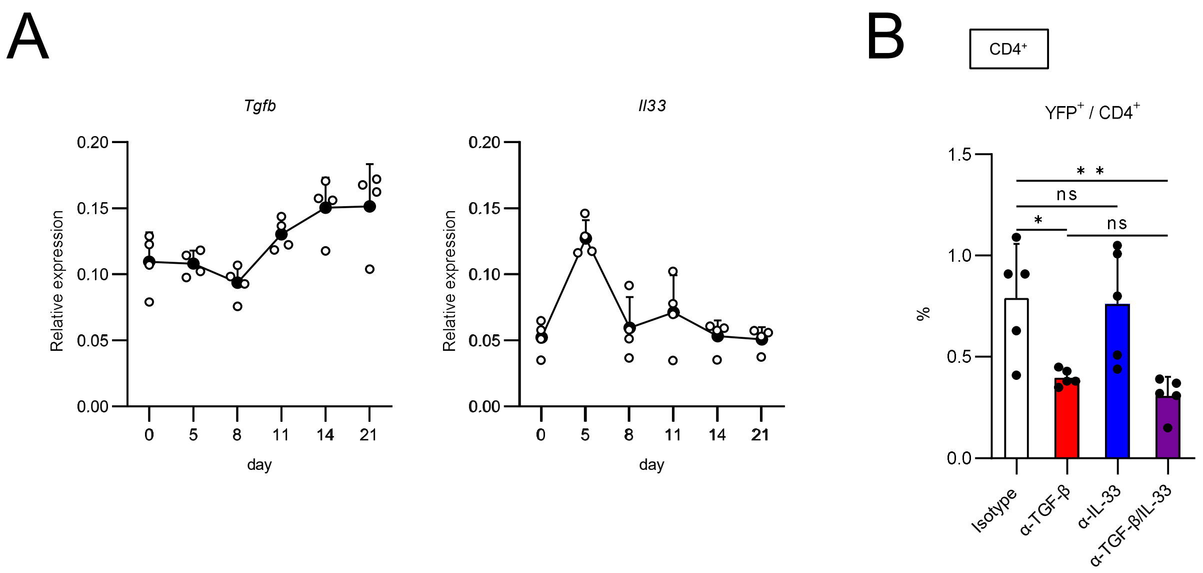

Supplementary Figure 4

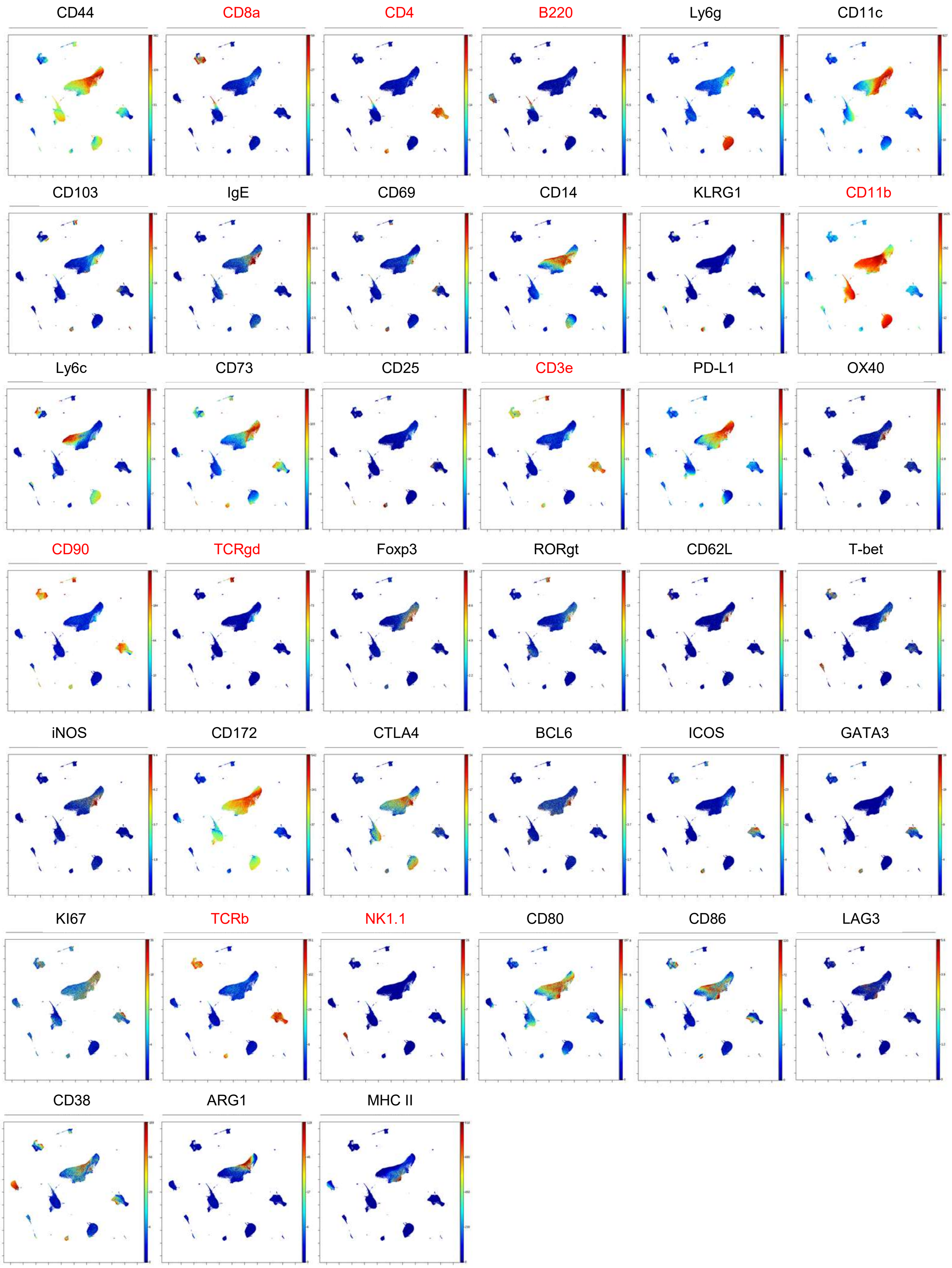

Supplement: Supplementary Figure 1 — Additional information of scRNA-seq analysis in Figure 1 (A) QC metrics for scRNA-seq datasets. Cells were filtered based on library size, number of expressed genes, and mitochondrial DNA content percentage. (B) UMAP plots of lung CD45+ cells indicating 19 clusters: whole (top), naïve group (bottom left) and BLM group (bottom right). (C) Heatmap indicating feature genes of 19 clusters. (D) UMAP plots indicating Cd3e expression. Based on Cd3e expression, clusters 4,8,9 and 11 were sub-cluster and further analyzed in Figures 1A–F (E) UMAP plots indicating Tff1 expression. [file DataSheet1.pdf]
